# Correction: Gq activity- and β-arrestin-1 scaffolding-mediated ADGRG2/CFTR coupling are required for male fertility

**DOI:** 10.7554/eLife.110882

**Published:** 2026-02-05

**Authors:** Dao-Lai Zhang, Yu-Jing Sun, Ming-Liang Ma, Yi-jing Wang, Hui Lin, Rui-Rui Li, Zong-Lai Liang, Yuan Gao, Zhao Yang, Dong-Fang He, Amy Lin, Hui Mo, Yu-Jing Lu, Mengjing Li, Wei Kong, Ka Young Chung, Fan Yi, Jian-Yuan Li, Ying-Ying Qin, Jingxin Li, Alex RB Thomsen, Alem W Kahsai, Zi-Jiang Chen, Zhi-Gang Xu, Mingyao Liu, Dali Li, Xiao Yu, Jin-Peng Sun

**Keywords:** Mouse

Zhang D-L, Sun Y-J, Ma M-L, Wang Y-, Lin H, Li R-R, Liang Z-L, Gao Y, Yang Z, He D-F, Lin A, Mo H, Lu Y-J, Li M-J, Kong W, Chung KY, Yi F, Li J-Y, Qin Y-Y, Li J, Thomsen ARB, Kahsai AW, Chen Z-J, Xu Z-G, Liu M, Li D, Yu X, Sun J-P. 2018. Gq activity- and β-arrestin-1 scaffolding-mediated ADGRG2/CFTR coupling are required for male fertility. *eLife* **7**:e33432. doi: 10.7554/eLife.33432.

Published 2 February 2018

A reader alerted us to inaccuracy in the representative efferent ductules images in Figure 2 and 3. This correction provides updates to Figure 3 and Figure 2 to improve anatomical accuracy. These changes affect only the specific panels noted below and do not alter the scientific conclusions of the study. The first author (Dao-Lai Zhang) assumes full responsibility for this matter.

For Figure 3A, the panels intended to represent efferent ductules in WT, *Adgrg2*^⁻/Y^, and *Gnaq*^+/^⁻ mice contained regions with both epididymis and efferent ductule tissues. During figure preparation, the complex anatomy of the murine male reproductive system and the small size of the efferent ductules led to the inclusion of mixed tissue regions in the selected images. To address this, we replaced the affected panels with accurately identified images that clearly and exclusively show efferent ductules, thereby improving the anatomical precision of the figure.

The corrected Figure 3 (updated for panel A) is shown here:

**Figure 1:**
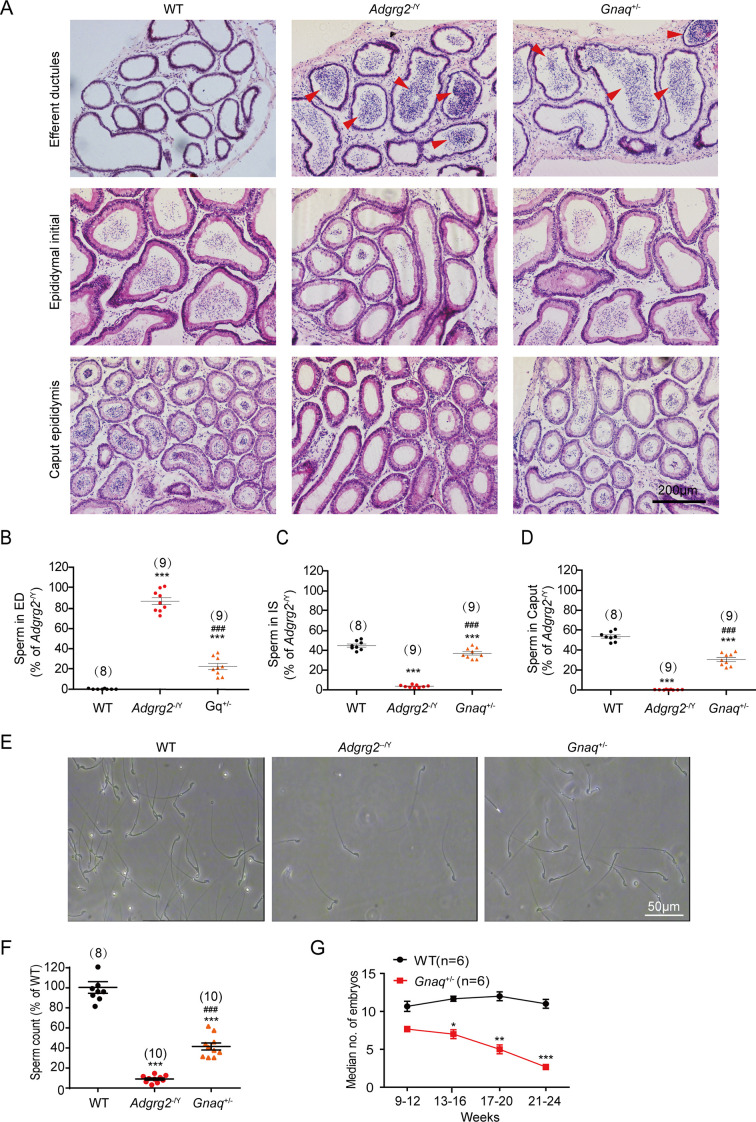


The originally published Figure 3 is shown for reference:

**Figure 2:**
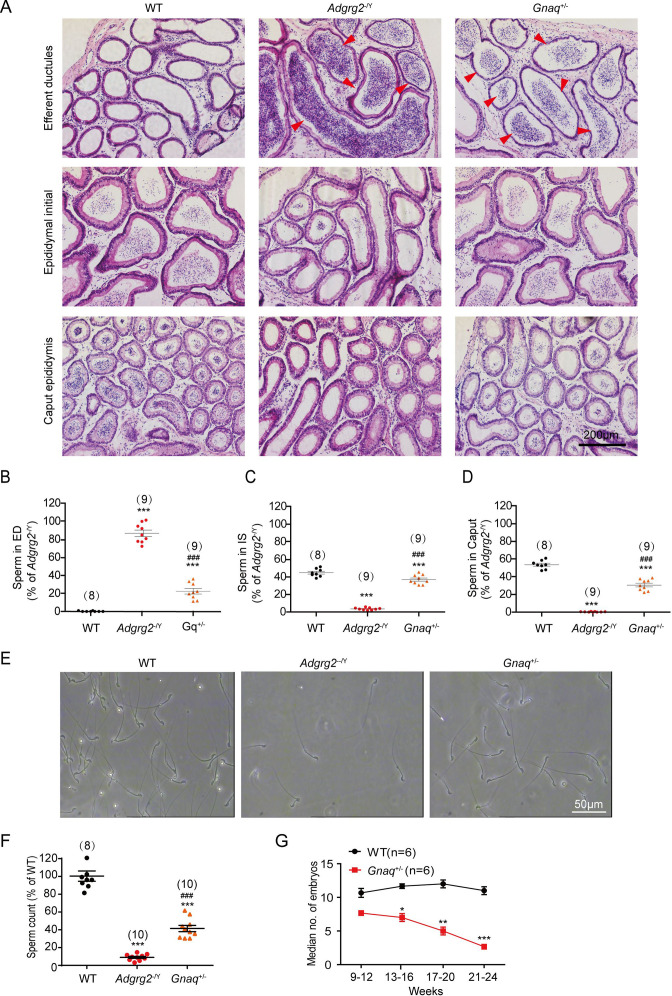


In Figure 2A, based on the guidance from experts during consultation and thorough re-examination of the original data, we identified that the originally published image of the wild-type (WT) efferent ductule did not clearly depict the typical anatomical features of proximal efferent ductules. We therefore replaced the image with a more appropriate representative showing the characteristic larger lumen diameter of proximal efferent ductules.

Additionally, the following explanatory note was added to the legend of Figure 2A to provide context for readers regarding lumen morphology variability: “Note that the WT lumen may have already collapsed due to the very rapid fluid reabsorption (Hess et al., 1997) by the time we took the photos.”

The legend for Figure 2A it shown for reference:“(A) Images of cultured ligated efferent ductules derived from WT male mice, *Adgrg2*^-/Y^ mice and *Gnaq*^+/-^ male mice. Ductule segments were selected by examination of the ciliary beat, which is a marker of cell integrity. Ductule pieces from *Adgrg2*^-/Y^, *Gnaq*^+/-^ or WT mice were ligated, microdissected and cultured for up to 72 hr. Scale bars, 200 μm. Note that the WT lumen may have already collapsed due to the very rapid fluid reabsorption (Hess et al., 1997) by the time we took the photos. ”

The corrected Figure 2 (updated for panel A) is shown here:

**Figure 3:**
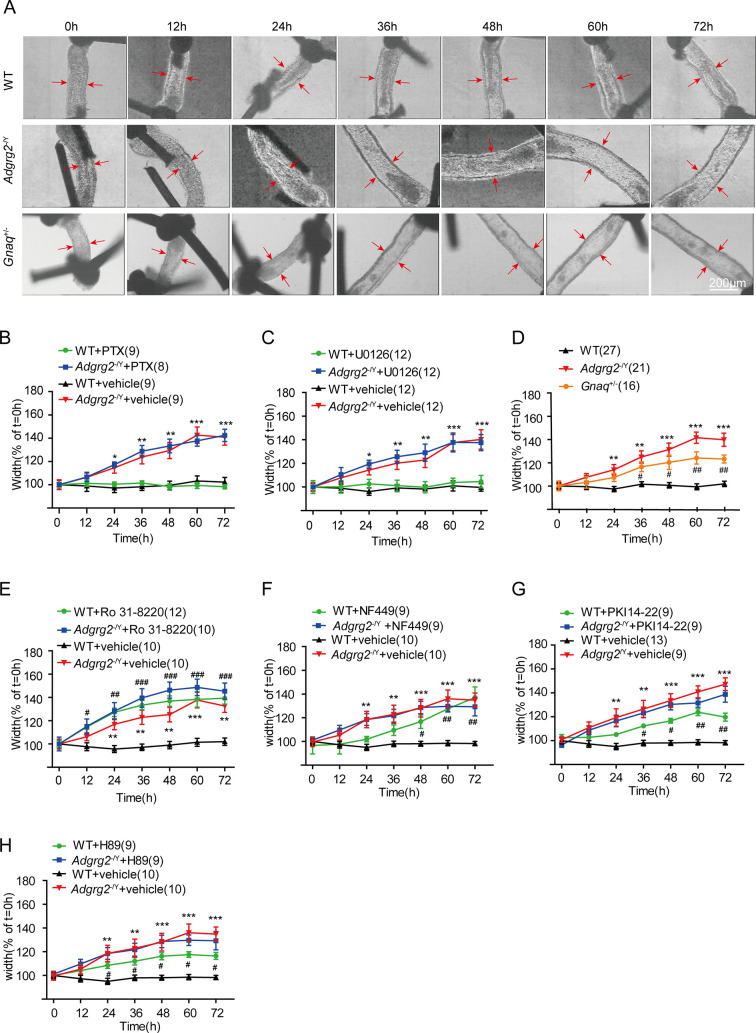


The originally published Figure 2 is shown for reference:

**Figure 4:**
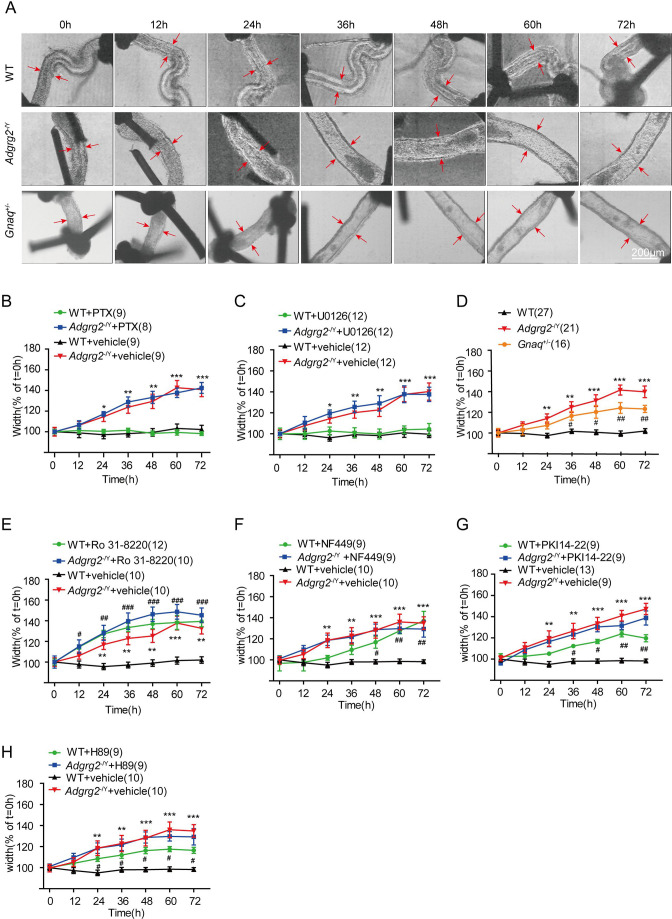


The article has been corrected accordingly.

