# Supplementary material for: Gq activity- and β-arrestin-1 scaffolding-mediated ADGRG2/CFTR coupling are required for male fertility
Source: eLife. 2018 Feb 2;7:e33432. doi: 10.7554/eLife.33432 (PMC5839696; doi:10.7554/eLife.33432)
Supplement: Supplementary file 1. [file elife-33432-supp1.doc]

**Supplementary File 1**

Primers for the Quantitative RT-PCR (qRT-PCR) analysis of mRNA transcription profiles of G protein subtypes and β-arrestins.

| primer name | Exon position | primer sequence |
| --- | --- | --- |
| Gαs-F | exon2 | ^2416^GCAGCGCGAGGCCAACAAAAA^2436^ |
| Gαs-R | exon3、exon4 | ^2591^GGTCCTCTTCGCCGCCCTCTC^2571^ |
| Gαolf-F | exon4 | ^784^ GTCTGGTTGACTACACACCCA^804^ |
| Gαolf-R | exon5 | ^970^GCCACGTAAATGATCGCAGTG^950^ |
| Gαi1-F | exon6、exon7 | ^962^ATGAACCGAATGCATGAG^979^ |
| Gαi1-R | exon8 | ^1189^GTCCTTCCTTTTATTGAGGTCT^1168^ |
| Gαi2-F | exon1 | ^167^GCGCTCTAAAATGATCGACAA^187^ |
| Gαi2-R | exon2 | ^270^TGGTGCTCTTCCCTGACTCT^251^ |
| Gαi3-F | exon6、exon7 | ^865^ATGAACCGAATGCATGAGAGCA^886^ |
| Gαi3-R | exon8 | ^1137^TTTGGTGTCAGTGGCACAGGTA^1116^ |
| Gαq-F | exon2 | ^687^GTCGGGCTACTCTGACGAAGA^707^ |
| Gαq-R | exon2、exon3 | ^827^TGTGCATGAGCCTTATTGTGTTC^805^ |
| Gα11-F | exon1 | ^182^CAACGCGGAGATCGAGAAACA^202^ |
| Gα11-R | exon2 | ^385^GCCTGCATGGCGGTAAAGAT^366^ |
| Gα12-F | exon1 | ^295^CGGCTGGTCAAGATCCTGC^313^ |
| Gα12-R | exon2 | ^461^GCGTCCACAAGAACCCTCG^443^ |
| Gα13-F | exon1 | ^270^GTCCAAGGAGATCGACAAATGC^291^ |
| Gα13-R | exon1、exon2 | ^478^CCAGCACCCTCATACCTTTGA^458^ |
| Gα15-F | exon2 | ^483^GATGAACGTACTCTTCCCGC^464^ |
| Gα15-R | exon1 | ^339^CAGAATCGACCAGGAGATCA^359^ |
| GαoA-F | exon8 | ^1546^CCCGTAGATTGTTGGCGATGA^1526^ |
| GαoA-R | exon7 | ^1224^CCGCATGCACGAGTCTCTCAT^1244^ |
| Gαt1-F | exon1 | ^104^AGAGGATGCTGAGAAGGATG^123^ |
| Gαt1-R | exon3 | ^315^ACTGAATGTTGAGCGTGGTC^296^ |
| Gαt2-F | exon3 | ^189^ TCCAAGGAGCTGGAGAAGAA^208^ |
| Gαt2-R | exon4 | ^345^ CTAGGCACTCTTCGGGTGAG ^326^ |
| GαZ-F | exon3 | ^1311^CTGCGCCTCTTTGACTCCAT^1330^ |
| GαZ-R | exon4 | ^1491^ACTGACGTTGGATGTAGACCG^1471^ |
| β-arrestin-1-F | exon5 | ^580^CACTGACACGGCTACAGGAG^599^ |
| β-arrestin-1-R | exon6 | ^682^TGCAGAGTGACTGAGCATGG^663^ |
| β-arrestin-2-F | exon8 | ^708^CACGCCACTTCCTCATGTCT^727^ |
| β-arrestin-2-R | exon9 | ^840^TTCTTGACGGTCTTGGCAGA^821^ |
| GAPDH-F  GAPDH-R | exon1  exon2 | ^780^GCCTTCCGTGTTCCTACC^797^  ^880^GCCTGCTTCACCACCTTC^863^ |
