# Supplementary material for: Gq activity- and β-arrestin-1 scaffolding-mediated ADGRG2/CFTR coupling are required for male fertility
Source: eLife. 2018 Feb 2;7:e33432. doi: 10.7554/eLife.33432 (PMC5839696; doi:10.7554/eLife.33432)
Supplement: Supplementary file 2. [file elife-33432-supp2.doc]

**Supplementary File 2**

Primers for the Quantitative RT-PCR (qRT-PCR) analysis of mRNA transcription profiles of potential osmotic drivers including selective ion channels and transporters.

| primer name | Exon position | primer sequence |
| --- | --- | --- |
| GPR64-F | exon12、exon13 | ^660^AGATTCGGCCAATGGAACAGT^680^ |
| GPR64-R | exon14 | ^807^GAAGACGATAACGGTGGG^787^ |
| NHE1-F | exon2 | ^1552^CATCCTTGTCTTCGGGGAGTC^1572^ |
| NHE1-R | exon3 | ^1744^GGAGGTGAAAGCTGCGATTAC^1724^ |
| NKCC-F | exon1 | ^773^TGCCCAGGATCGACCACTA^791^ |
| NKCC-R | exon2 | ^901^CTTCTCCATTCGCAAAGCCAT^881^ |
| DRA-F | exon8 | ^1137^GCCGTGGTTGGGAACATGA^1155^ |
| DRA-R | exon10 | ^1366^GCAAATCCTTTGAATGCTCCAG^1345^ |
| CFTR-F | exon12 | ^1779^GGTCAGCGTGCAAGGATTTCT^1799^ |
| CFTR-R | exon14 | ^1997^ACTGCCCTGATGCAAAATTAGT^1976^ |
| ANO1-F | exon5 | ^844^GAAAACCATCAACTCGGTTCTGC^866^ |
| ANO1-R | exon5、exon6 | ^970^GTCGAATAGGTGTTGCTTCTCC^949^ |
| SLC26A9-F | exon3 | ^288^TCCTGGCTCCCTAAATACAAGA^309^ |
| SLC26A9-R | exon4 | ^433^GGAGGAGTAGAGACCGTTGAC^411^ |
| V-ATPase-F | exon6 | ^818^ GAAGTTCAGCATGGTCCAAGTGTGG^842^ |
| V-ATPase-R | exon8 | ^1374^CATAGAAAGAAGCCAGCCGGGC^1353^ |
| CAII-F | exon2 | ^556^GCAGTGCTGAAAGGAGGACC^575^ |
| CAII-R | exon 4 | ^711^CCCATATTTGGTGTTCCAGTGAA^689^ |
| NHE3-F | exon11 | ^1650^CCTGGCCTTCATTCGCTCC^1668^ |
| NHE3-R | exon12 | ^1769^ACAGCACTGACATTTTCCCTC^1749^ |
| TRPC3-F | exon2 | ^503^ GCCTTCATGTTCGGTGCTC^521^ |
| TRPC3-R | exon4 | ^697^GGTCACCTCCAGATGCTCATT^677^ |
| CLCA1-F | exon12 | ^1829^CTAAACATCCGGTCTGCTAGACT^1851^ |
| CLCA1-R | exon13 | ^2037^ACCCGTGCGTACACAATCATC^2017^ |
| CAV1.2-F | exon1 | ^494^AATCACCCGAAGGGAGCAAG^513^ |
| CAV1.2-R | exon2 | ^612^AAGAAATGCAGCAACAGCCG^593^ |
| CAV1.3-F | exon2 | ^802^CCCAAAGAAAACGTCAGCAATAC^824^ |
| CAV1.3-R | exon3 | ^893^GAGGGATAAACAGAAAAGGGCA^872^ |
| CAV2.2-F | exon9 | ^1183^GATGGAGACTCGGACCAGAG^1202^ |
| CAV2.2-R  GAPDH-F  GAPDH-R | exon11  exon1  exon2 | ^1333^TGACCGTGAAACAGTTCTGCC^1313^  ^780^GCCTTCCGTGTTCCTACC^797^  ^880^GCCTGCTTCACCACCTTC^863^ |
