# Supplementary material for: Gq activity- and β-arrestin-1 scaffolding-mediated ADGRG2/CFTR coupling are required for male fertility
Source: eLife. 2018 Feb 2;7:e33432. doi: 10.7554/eLife.33432 (PMC5839696; doi:10.7554/eLife.33432)
Supplement: Supplementary file 3. [file elife-33432-supp3.doc]

**Supplementary File 3**

Primers for the construction of ADGRG2FL mutants (HM696AA, H696A, M697A, Y698A, K703A, V704A, F705A, Y708A, QL798AA, RK803EE).

| primer name | primer sequence |
| --- | --- |
| HM696AA-F | GGATTAGAAGCATTCGCCGCGTACCTAGCACTGG |
| HM696AA-R | CCAGTGCTAGGTACGCGGCGAATGCTTCTAATCC |
| H696A-F | GGGATTAGAAGCATTCGCCATGTACCTAGCACTG |
| H696A-R | CAGTGCTAGGTACATGGCGAATGCTTCTAATCCC |
| M697A-F | GATTAGAAGCATTCCACGCGTACCTAGCACTGGTC |
| M697A-R | GACCAGTGCTAGGTACGCGTGGAATGCTTCTAATC |
| Y698A-F | GAAGCATTCCACATGGCCCTAGCACTGGTCAAG |
| Y698A-R | CTTGACCAGTGCTAGGGCCATGTGGAATGCTTC |
| K703A-F | CATGTACCTAGCACTGGTCGCCGTGTTTAATACTTACATC |
| K703A-R | GATGTAAGTATTAAACACGGCGACCAGTGCTAGGTACATG |
| V704A-F | CTAGCACTGGTCAAGGCGTTTAATACTTACATC |
| V704A-R | GATGTAAGTATTAAACGCCTTGACCAGTGCTAG |
| F705A-F | CTAGCACTGGTCAAGGTGGCAAATACTTACATCCGAAAG |
| F705A-R | CTTTCGGATGTAAGTATTTGCCACCTTGACCAGTGCTAG |
| Y708A-F | GTCAAGGTGTTTAATACTGCCATCCGAAAGTACATCC |
| Y708A-R | GGATGTACTTTCGGATGGCAGTATTAAACACCTTGAC |
| QL798AA-F | GAATTAAAAAGAAGAAGGCAGCCGGAGCCCAGCGCAAAAC |
| QL798AA-R | GTTTTGCGCTGGGCTCCGGCTGCCTTCTTCTTTTTAATTC |
| RK803EE-F | CAGCTGGGAGCCCAGGAAGAAACTAGTATTCAAG |
| RK803EE-R | CTTGAATACTAGTTTCTTCCTGGGCTCCCAGCTG |
