# Supplementary material for: Gq activity- and β-arrestin-1 scaffolding-mediated ADGRG2/CFTR coupling are required for male fertility
Source: eLife. 2018 Feb 2;7:e33432. doi: 10.7554/eLife.33432 (PMC5839696; doi:10.7554/eLife.33432)
Supplement: Transparent reporting form [file elife-33432-transrepform.docx]

***eLife’s* transparent reporting form**

We encourage authors to provide detailed information *within their submission* to facilitate the interpretation and replication of experiments. If you have any questions, please contact us: [editorial@elifesciences.org](mailto:editorial@elifesciences.org).

**Sample-size estimation**

- You should state whether an appropriate sample size was computed when the study was being designed
- You should state the statistical method of sample size computation and any required assumptions
- If no explicit power analysis was used, you should describe how you decided what sample (replicate) size (number) to use

Please outline where this information can be found within the submission (e.g., page numbers or figure legends), or explain why this information doesn’t apply to your submission:

An appropriate sample size for all study were claimed in this manuscript submission.

For Figure 1A and 1D, the sample size (n) was shown in corresponding figures and the corresponding statements were descripted in figure legends of 1A and 1D at page 35.

For Figure 2B -2H, the sample size (n) was shown in corresponding figures and the corresponding statements were descripted in figure legends of 2B -2H at page 36.

For Figure 3B -3D, 3F-3G; the sample size (n) was shown in corresponding figures and the corresponding statements were descripted in figure legends of 13B -3D, 3F-3G at page 36-37.

For Figure 4A-4M the sample size (n) was shown in corresponding figures and the corresponding statements were descripted in figure legends of4A-4M at page 37-38.

For Figure 5A-5B the sample size (n) was shown in corresponding figures and the corresponding statements were descripted in figure legends of 5A-5B at page 38-39.

For Figure 6A-6B, 6D-6E the sample size (n) was shown in corresponding figures and the corresponding statements were descripted in figure legends of 6A-6B, 6D-6E at page 39.

For Figure 7A-7G the sample size (n) was shown in corresponding figures and the corresponding statements were descripted in figure legends of 7A-7G at page 40.

For Figure 8A,8C-8D, 8G-8H the sample size (n) was shown in corresponding figures and the corresponding statements were descripted in figure legends of 8A,8C-8D, 8G-8H at page 40-41.

For Figure 9A-9C the sample size (n) was shown in corresponding figures and the corresponding statements were descripted in figure legends of 9A-9C at page 42.

For Figure10B-10D the sample size (n) was shown in corresponding figures and the corresponding statements were descripted in figure legends of 10B-10D at page 42-43.

For Figure 11B-11D, 11E-11F the sample size (n) was shown in corresponding figures and the corresponding statements were descripted in figure legends of 11B-11D, 11E-11F at page 43.

For Figure 12C-12E the sample size (n) was shown in corresponding figures and the corresponding statements were descripted in figure legends of 12C-12E at page 44.

For Figure 13A-13F the sample size (n) was shown in corresponding figures and the corresponding statements were descripted in figure legends of 13A-13F at page 44-45.

The statistical method was described in methods section (page 29, line 872-880).

**Replicates**

- You should report how often each experiment was performed
- You should include a definition of biological versus technical replication
- The data obtained should be provided and sufficient information should be provided to indicate the number of independent biological and/or technical replicates
- If you encountered any outliers, you should describe how these were handled
- Criteria for exclusion/inclusion of data should be clearly stated
- High-throughput sequence data should be uploaded before submission, with a private link for reviewers provided (these are available from both GEO and ArrayExpress)

Please outline where this information can be found within the submission (e.g., page numbers or figure legends), or explain why this information doesn’t apply to your submission:

All number of replicates were shown as n number in the corresponding figure. These figures are following: Figure 1A, Figure 1D; Figure 2B-2H; Figure 3B-3D, Figure 3F-3G; Figure 4A-4M; Figure 5A-5B; Figure 6D-6E; Figure 7A-7G; Figure 8A; Figure 8C-8D, Figure 8G-8H; Figure 9A-9C, Figure 10B-10D; Figure 11B-11C,Figure 11E-11F; Figure 12C-12E; Figure 13A-13F

We have included the information for biological replication in corresponding figures in necessary.

We have not discarded any outlier data in our statistics.

**Statistical reporting**

- Statistical analysis methods should be described and justified
- Raw data should be presented in figures whenever informative to do so (typically when N per group is less than 10)
- For each experiment, you should identify the statistical tests used, exact values of N, definitions of center, methods of multiple test correction, and dispersion and precision measures (e.g., mean, median, SD, SEM, confidence intervals; and, for the major substantive results, a measure of effect size (e.g., Pearson's r, Cohen's d)
- Report exact p-values wherever possible alongside the summary statistics and 95% confidence intervals. These should be reported for all key questions and not only when the p-value is less than 0.05.

The statistical method was described in methods section (page 29, line 872-880).

Raw data of each measurement was shown in Figure 1A, Figure 1D; Figure 3B-3D, Figure 3F-3G; Figure 4A; Figure 5A-5B; Figure 6A-6B, Figure6E; Figure 7B-7C, Figure 7E, Figure 7G; Figure 8A, Figure 8D; Figure 8G-8H; Figure9C; Figure 10C-10D; and Figure 11B-11C, Figure 11F; Figure 12C-12E.

The statistical tests used, exact values of N, definitions of center, methods of multiple test correction, and dispersion and precision measures were described in corresponding figure legends.

We have reported every P-value in the corresponding figure legends.

For Figure 1A, 1D, the P-values were shown in figure legends of 1A, 1D at page 35.

For Figure 2B-2H, the P-values were shown in figure legends of 2B-2H at page 36.

For Figure 3B-3D, 3F-3G, the P-value was shown in figure legends3B-3D, 3F-3G at page 36-37.

For Figure 4A-4M, the P-values were shown in figure legends of 4A-4M at page 38.

For Figure 5A-5B, the P-values were shown in figure legends of 5A-5B at page 38.

For Figure 6E, the P-values were shown in figure legends of 6E at page 39.

For Figure 7B, 7E, 7G, the P-values were shown in figure legends of 7B, 7E, 7G, at page 40.

For Figure 8A, 8D, 8G-8H the P-values were shown in figure legends of 8A, 8D, 8G-8H, at page 41.

For Figure 9A-9C, the P-values were shown in figure legends of 9A-9C, at page 42.

For Figure 10C-10D, the P-values were shown in figure legends of 10C-10D, at page 43.

For Figure 11B-11C, 11F, the P-values were shown in figure legends of 11B-11C, 11F, at page 43.

For Figure 12C-12E, the P-values were shown in figure legends of 12C-12E, at page 44.

For Figure 13A-13F, the P-values were shown in figure legends of 13A-13F, at page 44-45.

Please outline where this information can be found within the submission (e.g., page numbers or figure legends), or explain why this information doesn’t apply to your submission:

(For large datasets, or papers with a very large number of statistical tests, you may upload a single table file with tests, Ns, etc., with reference to page numbers in the manuscript.)

**Additional data files (“source data”)**

- We encourage you to upload relevant additional data files, such as numerical data that are represented as a graph in a figure, or as a summary table
- Where provided, these should be in the most useful format, and they can be uploaded as “Source data” files linked to a main figure or table
- Include model definition files including the full list of parameters used
- Include code used for data analysis (e.g., R, MatLab)
- Avoid stating that data files are “available upon request”

Please indicate the figures or tables for which source data files have been provided:

We do not include any additional data in our current submission.
